# Supplementary material for: Development and Validation of a Novel Robot-Based Assessment of Upper Limb Sensory Processing in Chronic Stroke
Source: Brain Sci. 2022 Jul 29;12(8):1005. doi: 10.3390/brainsci12081005 (PMC9406163; doi:10.3390/brainsci12081005)
Supplement: Supplementary file 1 [file brainsci-12-01005-s001.zip › brainsci-1810947-supplementary.pdf]

Figure S1: Explored shapes (with starting point and direction indicated) and their corresponding visually presented options during the identification phase.

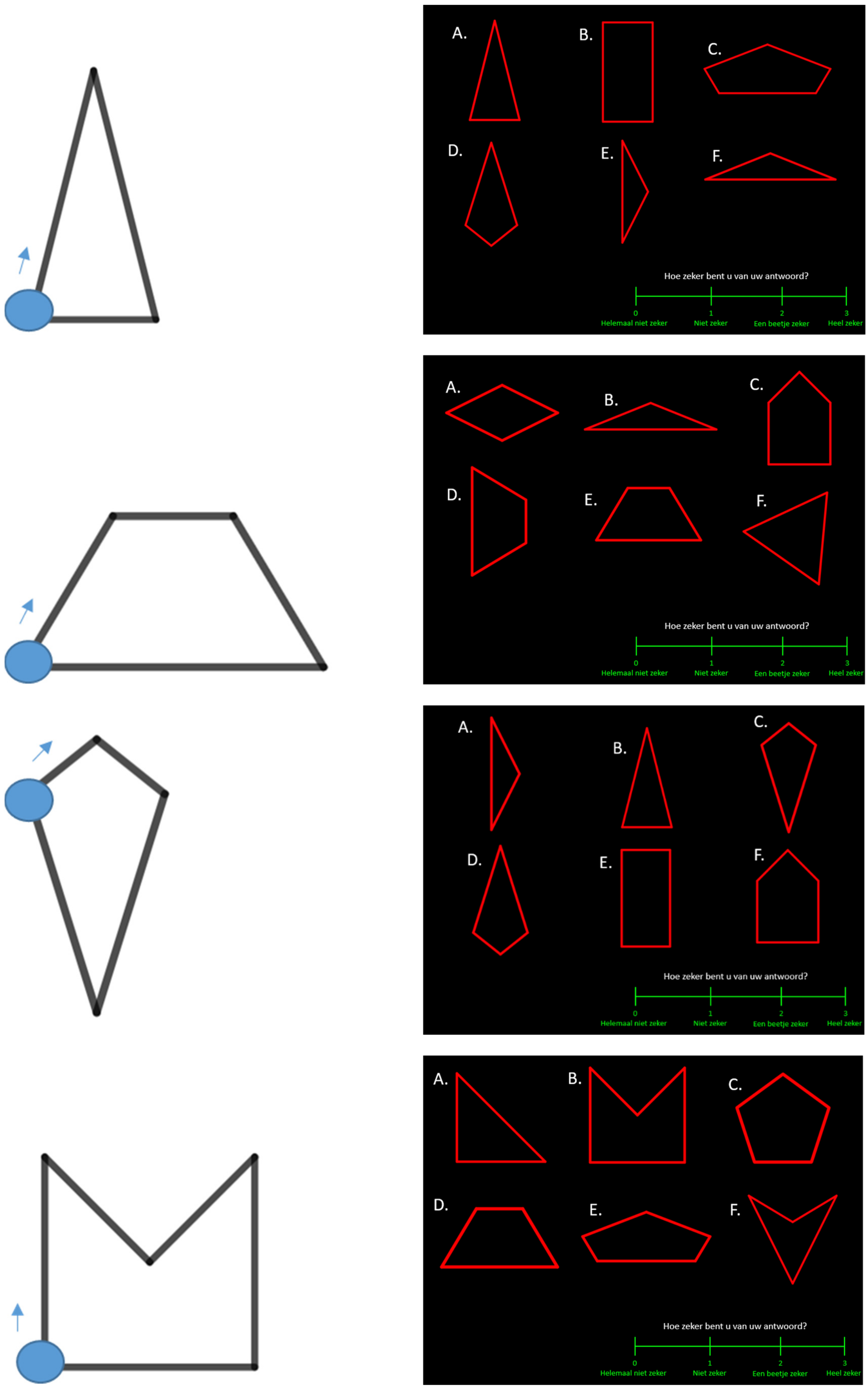

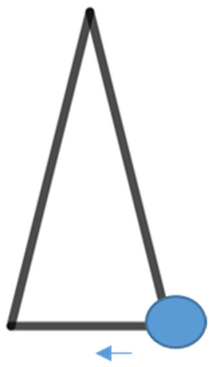

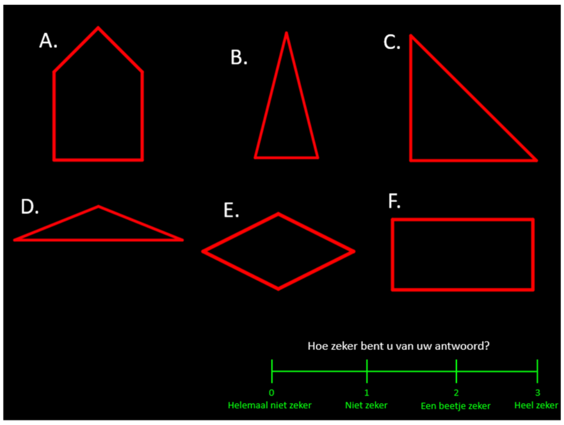
A.
B.
C.
D.
E.
F.
Hoe zeker bent u van uw antwoord?
0
Helemaal niet zeker
1
Niet zeker
2
Een beetje zeker
3
Heel zeker

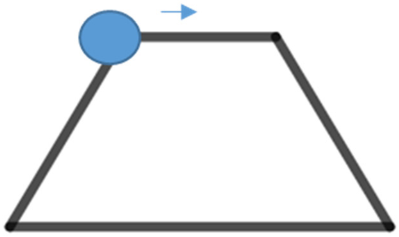

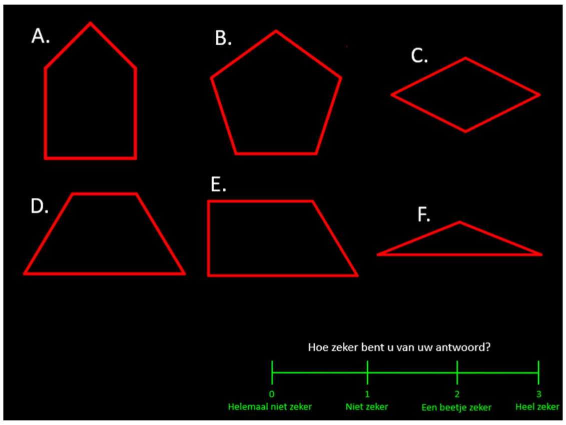
A.
B.
C.
D.
E.
F.
Hoe zeker bent u van uw antwoord?
0
Helemaal niet zeker
1
Niet zeker
2
Een beetje zeker
3
Heel zeker

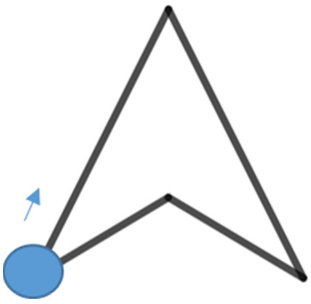

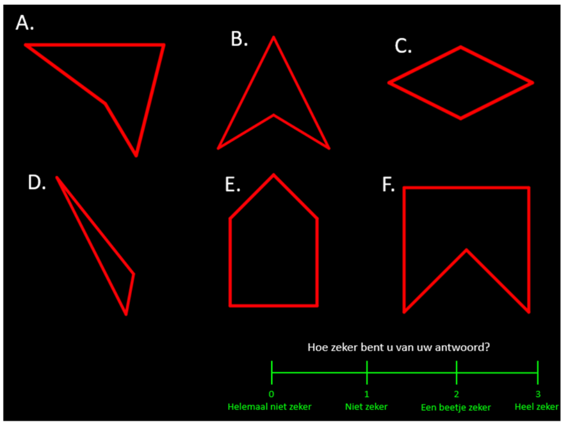
A.
B.
C.
D.
E.
F.
Hoe zeker bent u van uw antwoord?
0
Helemaal niet zeker
1
Niet zeker
2
Een beetje zeker
3
Heel zeker

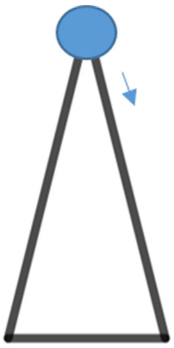

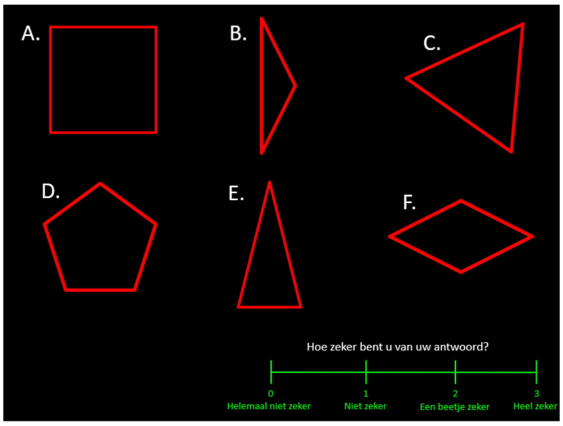
A.
B.
C.
D.
E.
F.
Hoe zeker bent u van uw antwoord?
0
Helemaal niet zeker
1
Niet zeker
2
Een beetje zeker
3
Heel zeker

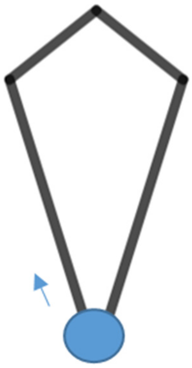

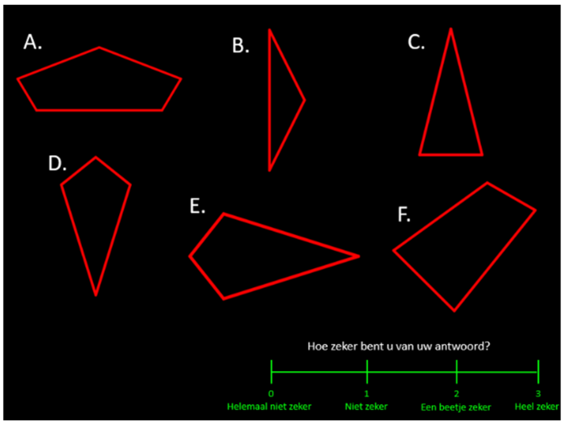
A.
B.
C.
D.
E.
F.
Hoe zeker bent u van uw antwoord?
0
Helemaal niet zeker
1
Niet zeker
2
Een beetje zeker
3
Heel zeker

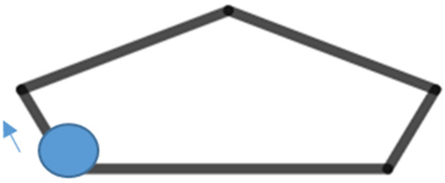

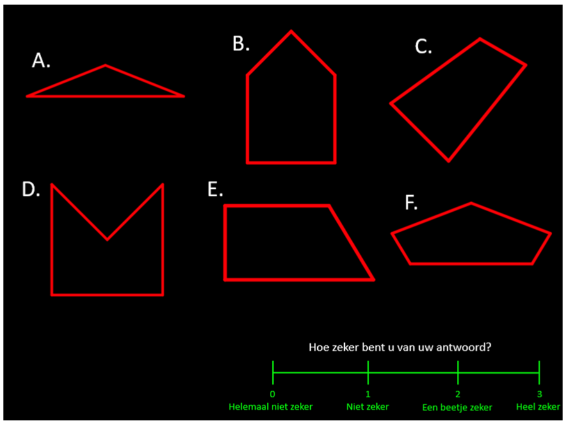
A.
B.
C.
D.
E.
F.
Hoe zeker bent u van uw antwoord?
0
Helemaal niet zeker
1
Niet zeker
2
Een beetje zeker
3
Heel zeker

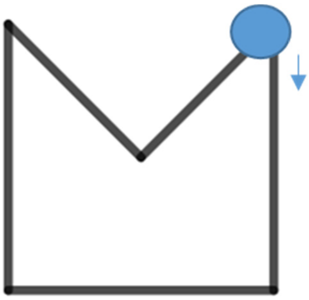

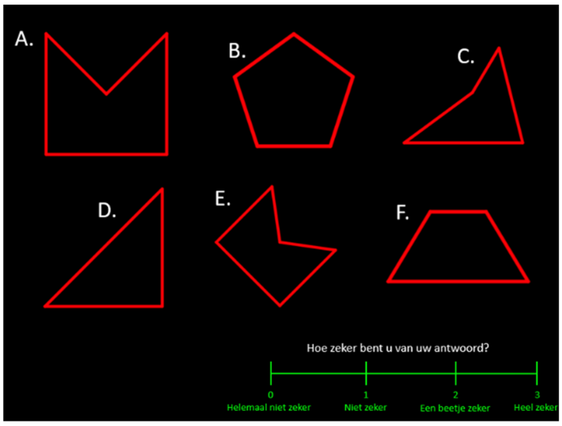
A.
B.
C.
D.
E.
F.
Hoe zeker bent u van uw antwoord?
0
Helemaal niet zeker
1
Niet zeker
2
Een beetje zeker
3
Heel zeker

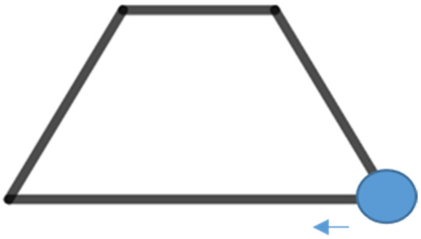

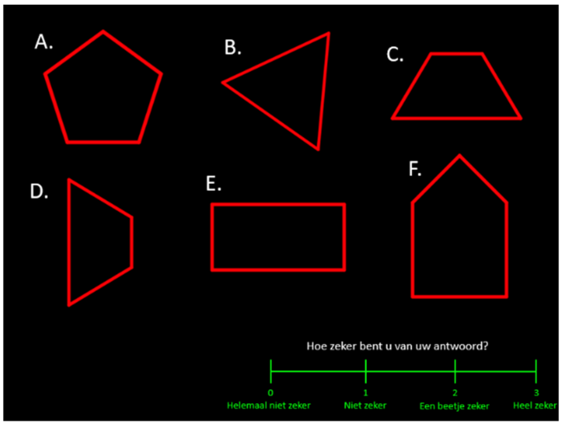
A.
B.
C.
D.
E.
F.
Hoe zeker bent u van uw antwoord?
0
Helemaal niet zeker
1
Niet zeker
2
Een beetje zeker
3
Heel zeker

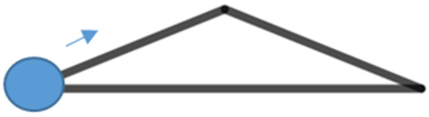

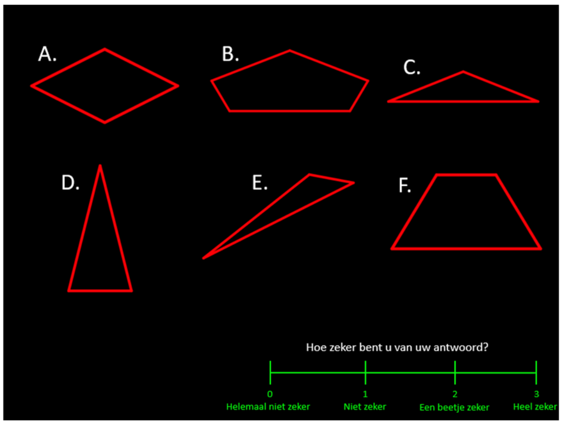
A.
B.
C.
D.
E.
F.
Hoe zeker bent u van uw antwoord?
0 Helemaal niet zeker
1 Niet zeker
2 Een beetje zeker
3 Heel zeker

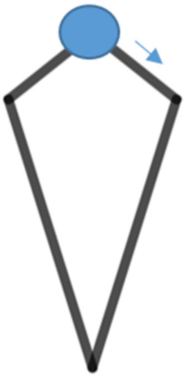

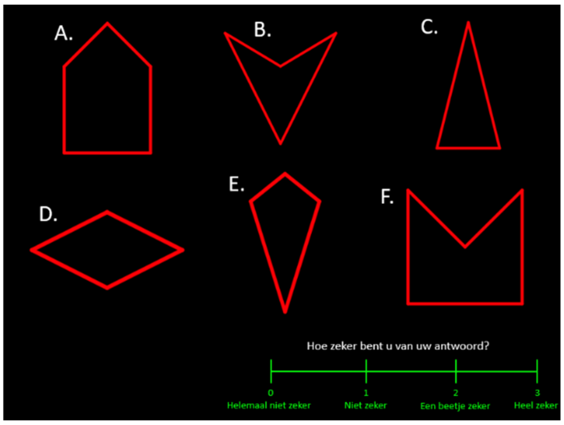
A.
B.
C.
D.
E.
F.
Hoe zeker bent u van uw antwoord?
0 Helemaal niet zeker
1 Niet zeker
2 Een beetje zeker
3 Heel zeker

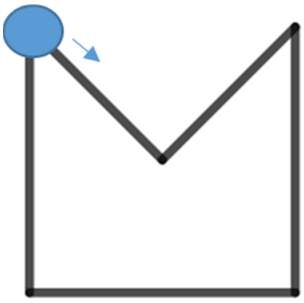

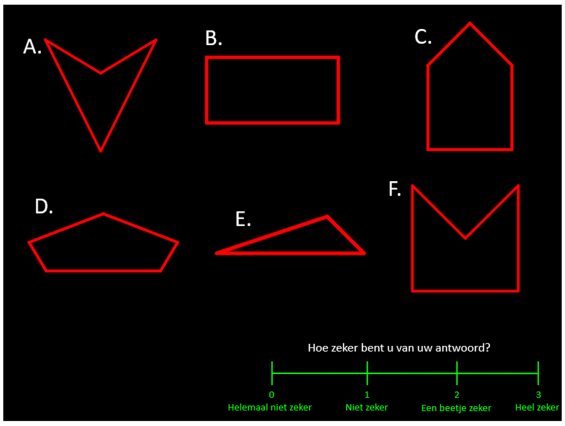
A.
B.
C.
D.
E.
F.
Hoe zeker bent u van uw antwoord?
0 Helemaal niet zeker
1 Niet zeker
2 Een beetje zeker
3 Heel zeker

Table S1: Correlation coefficients between reproduction and identification parameters of the robot-based passive and active sensory processing tasks, and clinical and robot-based assessments of somatosensory function, motor function, cognitive function and activities.

		Cross-correlation X Passive task		Cross-correlation X Active task	
		rw	95% CI	rw	95% CI
Somatosensory function	EmNSA-SB total score	0.13	(−0.33 0.54)	0.39	(−0.06 0.71)
	EmNSA total score	0.21	(−0.25 0.60)	0.47	(0.03 0.75)
	st-NSA total score	0.30	(−0.17 0.65)	0.45	(0.00 0.74)
	PTT average	0.27	(−0.19 0.64)	0.04	(−0.41 0.47)
	TDT total score	0.52	(0.09 0.78)	0.62	(0.24 0.83)
	TDT area under the curve	0.58	(0.18 0.81)	0.71	(0.38 0.87)
	WPST average error	−0.29	(−0.65 0.18)	−0.40	(−0.71 0.05)
	fTORT total score	0.31	(−0.15 0.66)	0.32	(−0.15 0.67)
	APM affected arm task score	−0.27	(−0.64 0.19)	−0.45	(−0.74 −0.01)
Motor function	FM-UE total score	0.47	(0.03 0.75)	0.63	(0.26 0.84)
	VGR affected arm task score	−0.39	(−0.71 0.07)	−0.64	(−0.84 −0.27)
	VGR less affected arm task score	−0.10	(−0.52 0.35)	−0.23	(−0.61 0.23)
	VGR inter-limb task score	−0.44	(−0.74 0.00)	−0.67	(−0.86 −0.33)
Cognitive function	MoCA total score	−0.15	(−0.55 0.32)	−0.36	(−0.69 0.09)
Activities	ARAT total score	0.37	(−0.09 0.70)	0.56	(0.16 0.80)

BI total score	0.17	(−0.29 0.57]	0.42	(−0.03 0.73)

Red = low correlation (rw = 0.30-0.50); Yellow = moderate correlation (rw = 0.50-0.70); Green = high correlation (rw > 0.70).

Note that lower values are associated with better performance for PTT, WPST, APM and VGR. For all other outcomes, higher values are associated with better performance.

Abbreviations: r_W = Winsorized correlation coefficient; CI = confidence interval; EmNSA-SB = sharp-blunt discrimination subscale of Erasmus modified Nottingham sensory assessment; EmNSA = Erasmus modified Nottingham sensory assessment; st-NSA = stereognosis section of original Nottingham sensory assessment; PTT = perceptual threshold of touch; TDT = tactile discrimination test; WPST = wrist position sense test; fTORT = functional tactile object recognition test; APM = arm position matching; FM-UE = Fugl-Meyer upper extremity assessment; VGR = visually guided reaching; MoCA = Montreal cognitive assessment; ARAT = action research arm rest; BI = Barthel index.

		Cross-correlation Y Passive task		Cross-correlation Y Active task	
		rw	95% CI	rw	95% CI
Somatosensory function	EmNSA-SB total score	0.37	(−0.08 0.70)	0.36	(−0.10 0.69)
	EmNSA total score	0.35	(−0.11 0.69)	0.37	(−0.08 0.70)
	st-NSA total score	0.18	(−0.29 0.58)	0.30	(−0.16 0.66)
	PTT average	0.23	(−0.23 0.61)	0.29	(−0.18 0.65)
	TDT total score	0.45	(0.01 0.74)	0.59	(0.20 0.82)
	TDT area under the curve	0.49	(0.06 0.77)	0.61	(0.23 0.83)
	WPST average error	−0.37	(−0.70 0.09)	−0.55	(−0.80 −0.14)
	fTORT total score	0.40	(−0.05 0.72)	0.41	(−0.04 0.72)
	APM affected arm task score	−0.22	(−0.61 0.24)	−0.23	(−0.61 0.23)
Motor function	FM-UE total score	0.40	(−0.06 0.71)	0.48	(0.05 0.76)
	VGR affected arm task score	−0.47	(−0.75 -0.03)	−0.43	(−0.73 0.01)
	VGR less affected arm task score	−0.12	(−0.53 0.34)	−0.12	(−0.53 0.34)
	VGR inter-limb task score	−0.48	(−0.76 -0.05)	−0.47	(−0.76 −0.04)
Cognitive function	MoCA total score	−0.14	(−0.55 0.32)	−0.19	(−0.58 0.27)
Activities	ARAT total score	0.34	(−0.12 0.68)	0.38	(−0.07 0.71)
	BI total score	0.15	(−0.31 0.56)	0.29	(−0.18 0.65)

Red = low correlation (rw = 0.30-0.50); Yellow = moderate correlation (rw = 0.50-0.70); Green = high correlation (rw > 0.70).

Note that lower values are associated with better performance for PTT, WPST, APM and VGR. For all other outcomes, higher values are associated with better performance.

Abbreviations: r_W = Winsorized correlation coefficient; CI = confidence interval; EmNSA-SB = sharp-blunt discrimination subscale of Erasmus modified Nottingham sensory assessment; EmNSA = Erasmus modified Nottingham sensory assessment; st-NSA = stereognosis section of original Nottingham sensory assessment; PTT = perceptual threshold of touch; TDT = tactile discrimination test; WPST = wrist position sense test; fTORT = functional tactile object recognition test; APM = arm position matching; FM-UE = Fugl-Meyer upper extremity assessment; VGR = visually guided reaching; MoCA = Montreal cognitive assessment; ARAT = action research arm rest; BI = Barthel index.

		DTW Passive task		DTW Active task	
		rw	95% CI	rw	95% CI
Somatosensory function	EmNSA-SB total score	−0.24	(−0.62 0.22)	−0.22	(−0.61 0.24)
	EmNSA total score	−0.31	(−0.66 0.15)	−0.28	(−0.64 0.18)
	st-NSA total score	−0.21	(−0.60 0.26)	−0.17	(−0.57 0.29)
	PTT average	−0.34	(−0.68 0.12)	−0.07	(−0.49 0.39)
	TDT total score	−0.32	(−0.67 0.14)	−0.43	(−0.74 0.01)
	TDT area under the curve	−0.26	(−0.63 0.20)	−0.43	(−0.73 0.01)
	WPST average error	0.34	(−0.12 0.68)	0.39	(−0.06 0.71)
	fTORT total score	0.04	(−0.41 0.48)	−0.18	(−0.58 0.28)
	APM affected arm task score	0.00	(−0.44 0.44)	0.24	(−0.23 0.61)
Motor function	FM-UE total score	−0.23	(−0.61 0.23)	−0.39	(−0.71 0.06)
	VGR affected arm task score	0.33	(−0.13 0.68)	0.48	(0.05 0.76)
	VGR less affected arm task score	−0.10	(−0.52 0.36)	−0.11	(−0.53 0.35)
	VGR inter-limb task score	0.35	(−0.11 0.69)	0.54	(0.13 0.79)
Cognitive function	MoCA total score	0.15	(−0.31 0.56)	0.15	(−0.32 0.55)
Activities	ARAT total score	−0.17	(−0.57 0.30)	−0.33	(−0.68 0.13)
	BI total score	−0.27	(−0.64 0.19)	−0.56	(−0.80 −0.15)

Red = low correlation (rw = 0.30-0.50); Yellow = moderate correlation (rw = 0.50-0.70); Green = high correlation (rw > 0.70).

Note that lower values are associated with better performance for DTW, PTT, WPST, APM and VGR. For all other outcomes, higher values are associated with better performance.

Abbreviations: DTW = dynamic time warping; r_W = Winsorized correlation coefficient; CI = confidence interval; EmNSA-SB = sharp-blunt discrimination subscale of Erasmus modified Nottingham sensory assessment; EmNSA = Erasmus modified Nottingham sensory assessment; st-NSA = stereognosis section of original Nottingham sensory assessment; PTT = perceptual threshold of touch; TDT = tactile discrimination test; WPST = wrist position sense test; fTORT = functional tactile object recognition test; APM = arm position matching; FM-UE = Fugl-Meyer upper extremity assessment; VGR = visually guided reaching; MoCA = Montreal cognitive assessment; ARAT = action research arm rest; BI = Barthel index.

		Procrustes Passive task		Procrustes Active task	
		rw	95% CI	rw	95% CI
Somatosensory function	EmNSA-SB total score	−0.40	(−0.71 0.06)	−0.43	(−0.74 0.01)
	EmNSA total score	−0.42	(−0.73 0.03)	−0.48	(−0.76 -0.05)
	st-NSA total score	−0.04	(−0.48 0.41)	−0.22	(−0.60 0.25)
	PTT average	−0.24	(−0.62 0.22)	−0.17	(−0.57 0.30)
	TDT total score	−0.45	(−0.74 −0.01)	−0.62	(−0.84 −0.25)
	TDT area under the curve	−0.46	(−0.75 −0.02)	−0.68	(−0.86 −0.34)
	WPST average error	0.28	(−0.19 0.64)	0.35	(−0.11 0.69)
	fTORT total score	−0.38	(−0.70 0.07)	−0.34	(−0.68 0.12)
	APM affected arm task score	0.12	(−0.34 0.53)	0.33	(−0.13 0.67)
Motor function	FM-UE total score	−0.25	(−0.62 0.22)	−0.51	(−0.77 −0.08)
	VGR affected arm task score	0.38	(−0.08 0.70)	0.54	(0.13 0.79)
	VGR less affected arm task score	0.01	(−0.44 0.45)	0.11	(−0.35 0.53)
	VGR inter-limb task score	0.43	(−0.02 0.73)	0.61	(0.23 0.83)
Cognitive function	MoCA total score	0.05	(−0.40 0.48)	0.32	(−0.15 0.67)
Activities	ARAT total score	−0.20	(−0.59 0.27)	−0.44	(−0.74 0.00)
	BI total score	−0.18	(−0.58 0.28)	−0.40	(−0.71 0.05)

Red = low correlation (rw = 0.30-0.50); Yellow = moderate correlation (rw = 0.50-0.70); Green = high correlation (rw > 0.70).

Note that lower values are associated with better performance for Procrustes, PTT, WPST, APM and VGR. For all other outcomes, higher values are associated with better performance.

Abbreviations: r_W = Winsorized correlation coefficient; CI = confidence interval; EmNSA-SB = sharp-blunt discrimination subscale of Erasmus modified Nottingham sensory assessment; EmNSA = Erasmus modified Nottingham sensory assessment; st-NSA = stereognosis section of original Nottingham sensory assessment; PTT = perceptual threshold of touch; TDT = tactile discrimination test; WPST = wrist position sense test; fTORT = functional tactile object recognition test; APM = arm position matching; FM-UE = Fugl-Meyer upper extremity assessment; VGR = visually guided reaching; MoCA = Montreal cognitive assessment; ARAT = action research arm rest; BI = Barthel index.

		% identified Passive task		% identified Active task	
		rw	95% CI	rw	95% CI
Somatosensory function	EmNSA-SB total score	0.43	(−0.01 0.73)	0.18	(−0.29 0.58)
	EmNSA total score	0.39	(−0.07 0.71)	0.42	(−0.02 0.73)
	st-NSA total score	−0.05	(−0.48 0.40)	0.35	(−0.11 0.69)
	PTT average	0.21	(−0.26 0.60)	0.08	(−0.37 0.51)
	TDT total score	0.55	(0.14 0.80)	0.61	(0.23 0.83)
	TDT area under the curve	0.50	(0.08 0.77)	0.60	(0.21 0.82)
	WPST average error	−0.46	(−0.75 −0.02)	−0.23	(−0.61 0.23)
	fTORT total score	0.23	(−0.24 0.61)	0.32	(−0.14 0.67)
	APM affected arm task score	−0.12	(−0.53 0.34)	−0.39	(−0.71 0.07)
Motor function	FM-UE total score	0.23	(−0.23 0.61)	0.41	(−0.05 0.72)
	VGR affected arm task score	−0.29	(−0.65 0.17)	−0.34	(−0.68 0.12)
	VGR less affected arm task score	−0.01	(−0.45 0.44)	0.29	(−0.17 0.65)
	VGR inter-limb task score	−0.36	(−0.69 0.10)	−0.49	(−0.77 −0.07)
Cognitive function	MoCA total score	−0.24	(−0.62 0.23)	−0.19	(−0.58 0.28)
Activities	ARAT total score	0.19	(−0.28 0.58)	0.34	(−0.12 0.68)
	BI total score	0.31	(−0.16 0.66)	0.30	(−0.16 0.66)

Red = low correlation (rw = 0.30-0.50); Yellow = moderate correlation (rw = 0.50-0.70); Green = high correlation (rw > 0.70).

Note that lower values are associated with better performance for PTT, WPST, APM and VGR. For all other outcomes, higher values are associated with better performance.

Abbreviations: r_W = Winsorized correlation coefficient; CI = confidence interval; EmNSA-SB = sharp-blunt discrimination subscale of Erasmus modified Nottingham sensory assessment; EmNSA = Erasmus modified Nottingham sensory assessment; st-NSA = stereognosis section of original Nottingham sensory assessment; PTT = perceptual threshold of touch; TDT = tactile discrimination test; WPST = wrist position sense test; fTORT = functional tactile object recognition test; APM = arm position matching; FM-UE = Fugl-Meyer upper extremity assessment; VGR = visually guided reaching; MoCA = Montreal cognitive assessment; ARAT = action research arm rest; BI = Barthel index.

Figure S2: Scatterplots of clinical and robot-based assessments of somatosensory function, motor function, cognitive function and activities against all parameters of the robot-based passive and active sensory processing tasks.

Erasmus modified Nottingham sensory assessment – sharp-blunt discrimination subscale

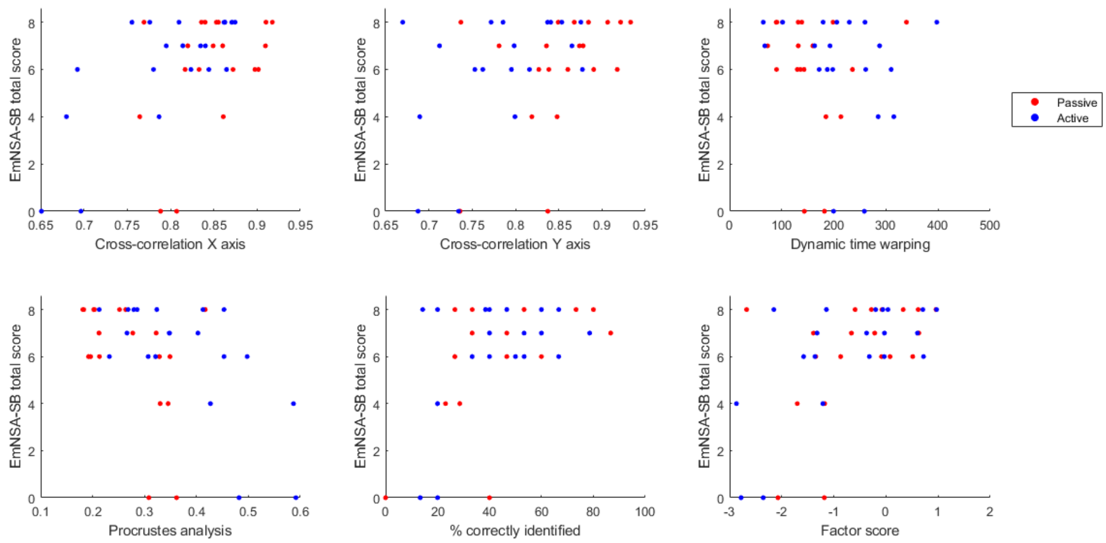

Erasmus modified Nottingham sensory assessment

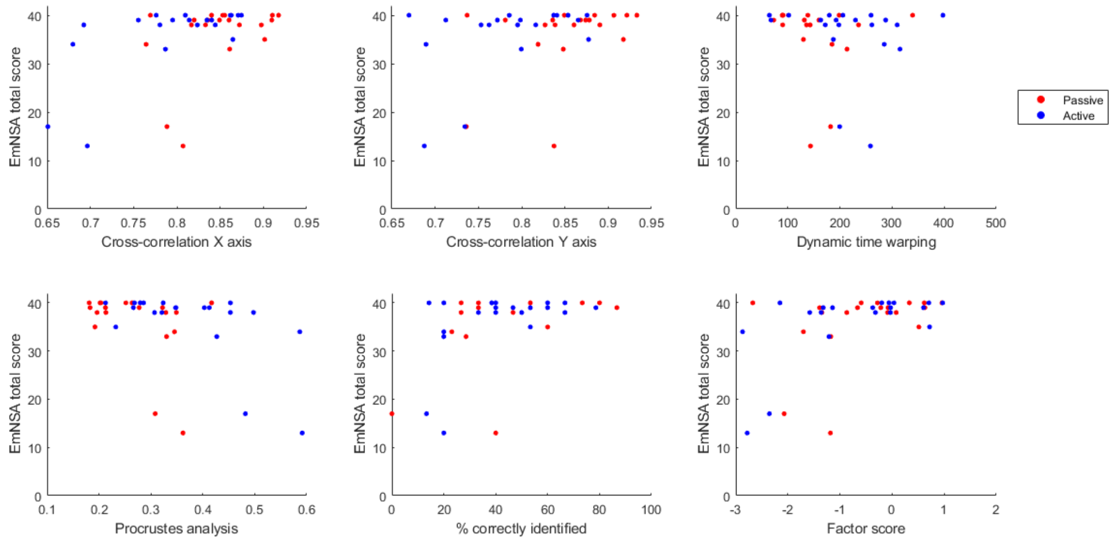

Stereognosis section of original Nottingham sensory assessment

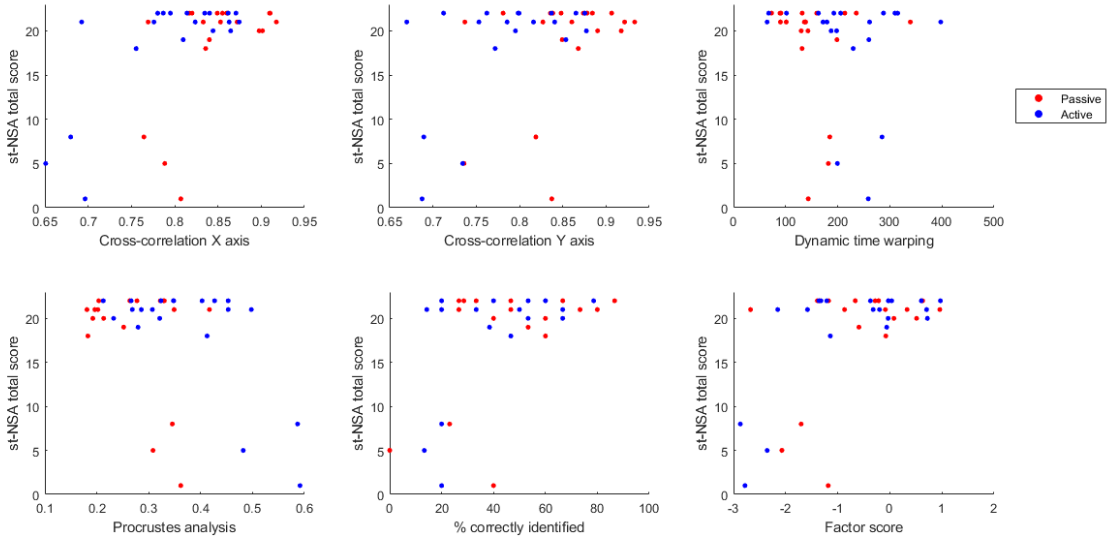

Perceptual threshold of touch

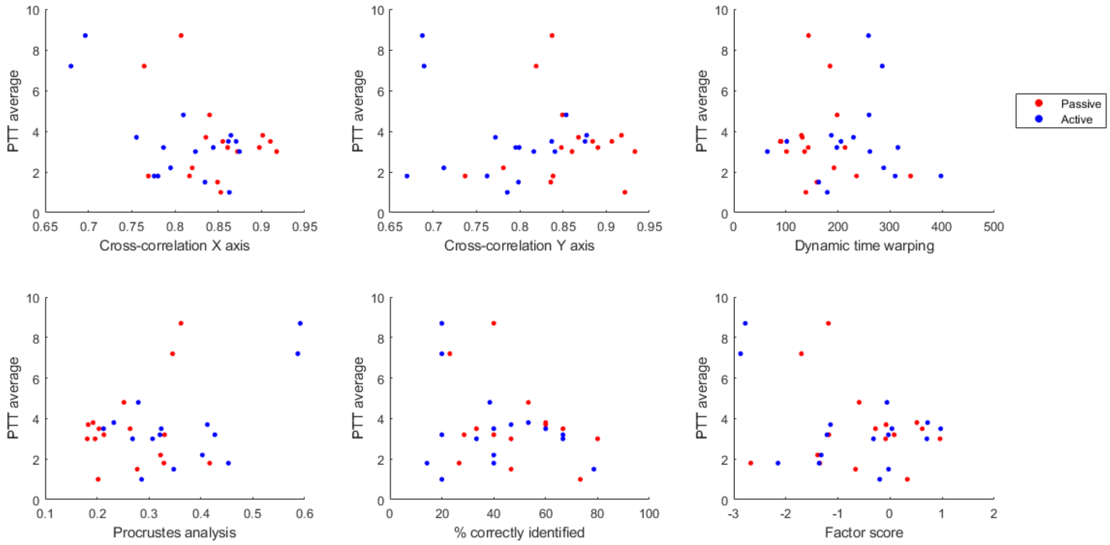

Tactile discrimination test

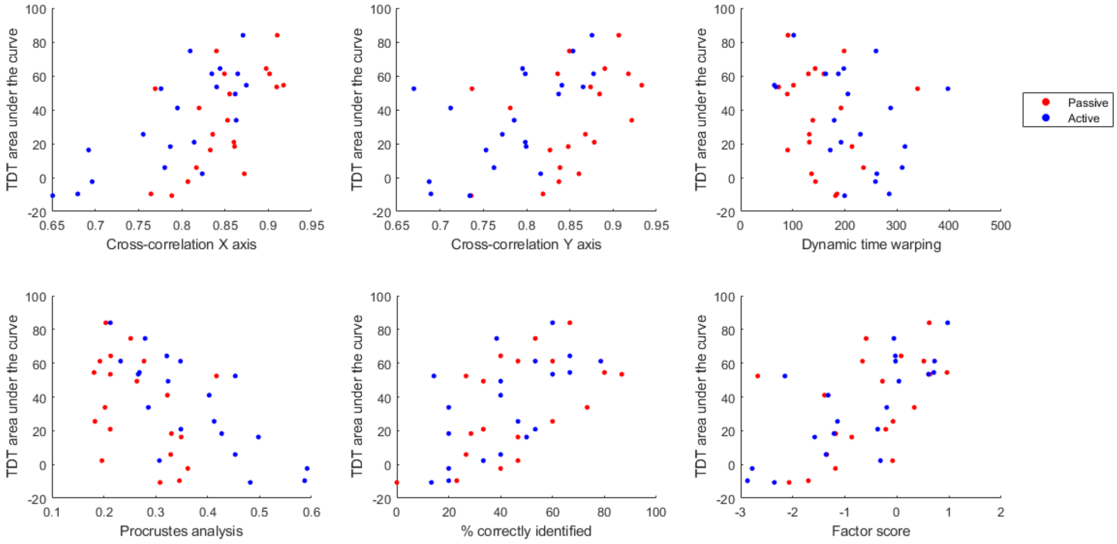

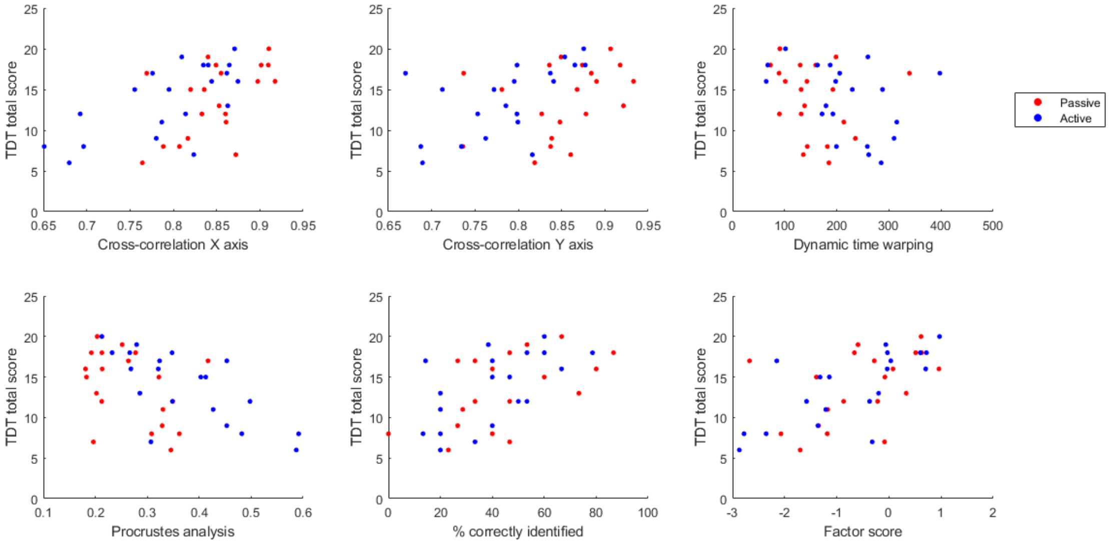

Wrist position sense test

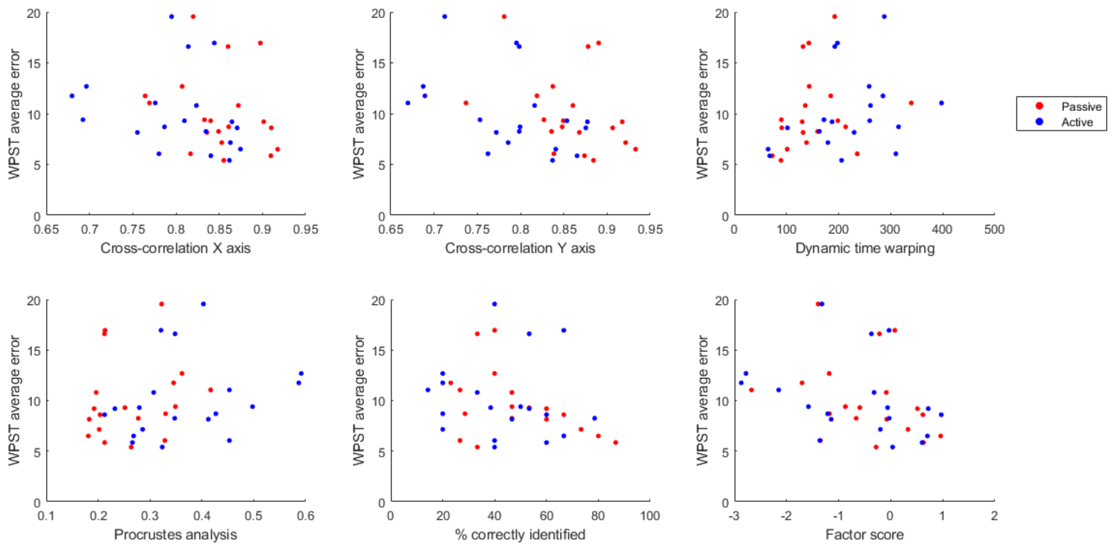

Functional tactile object recognition test

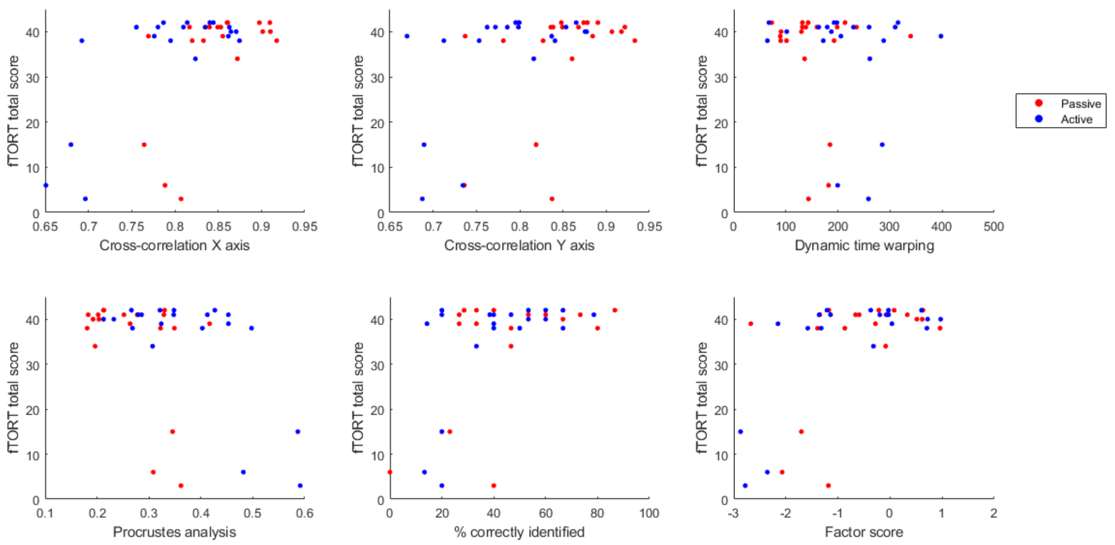

Arm position matching

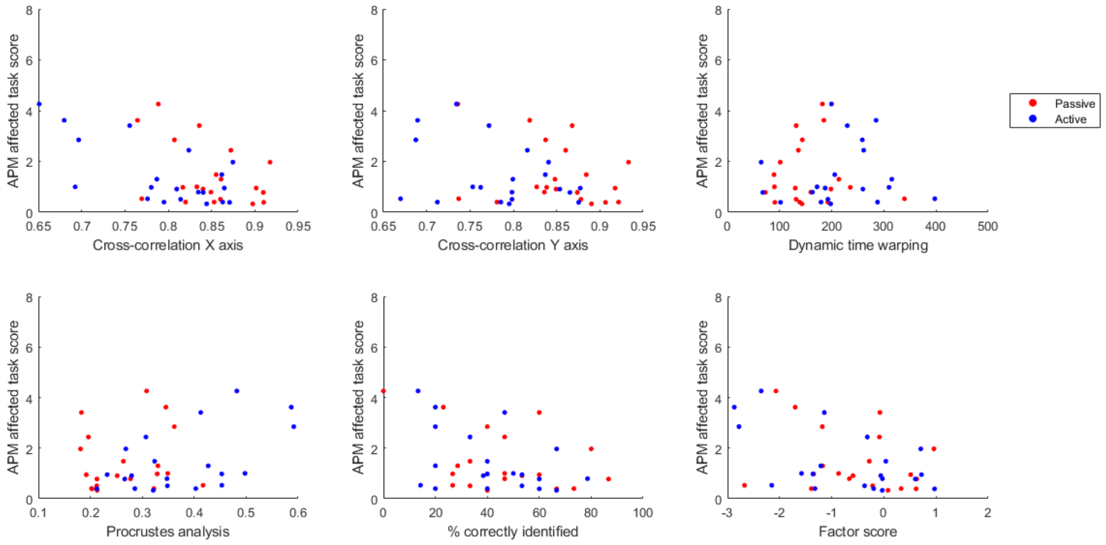

Fugl-Meyer upper extremity assessment

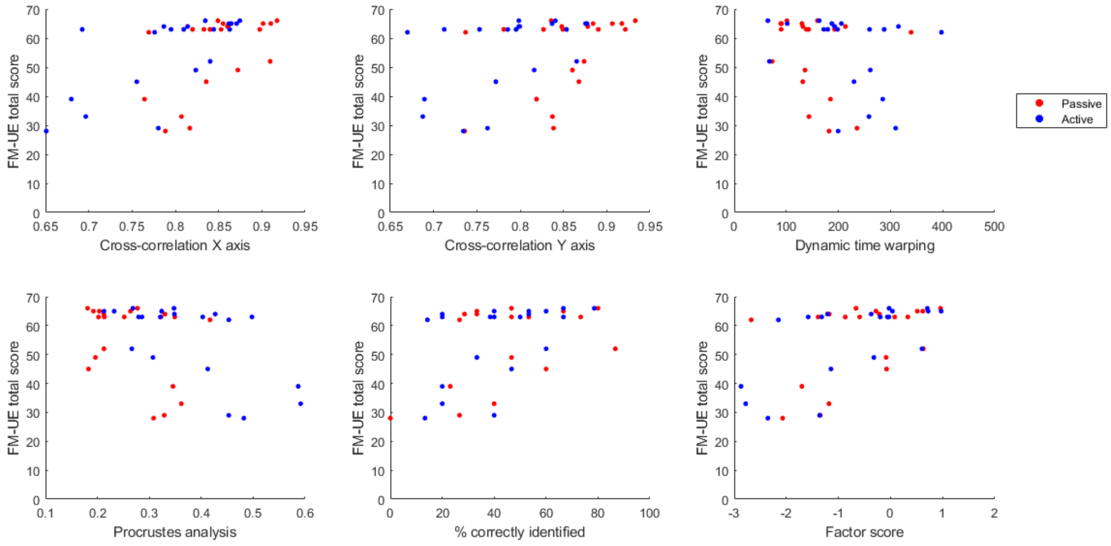

Visually guided reaching

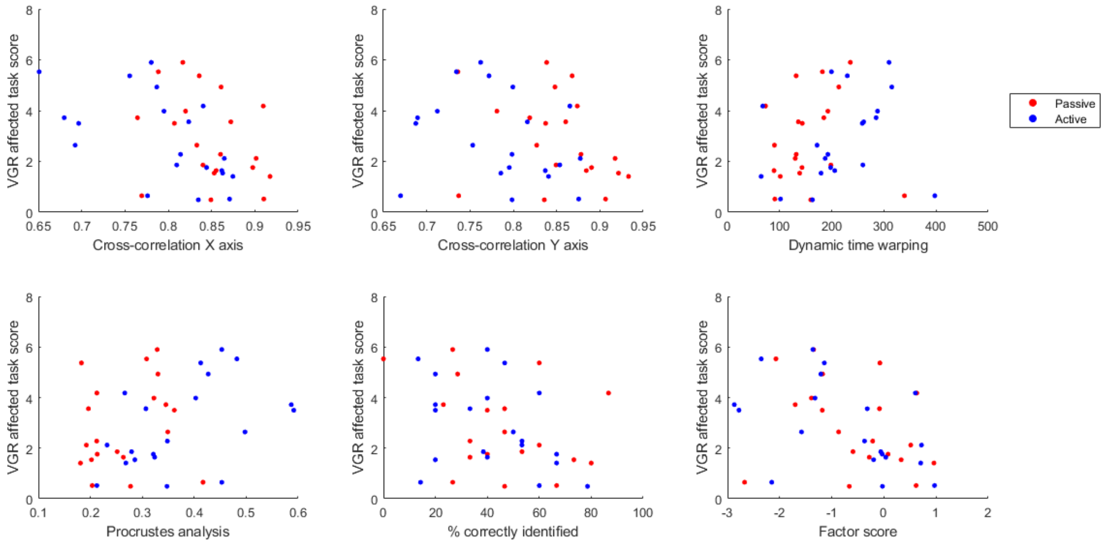

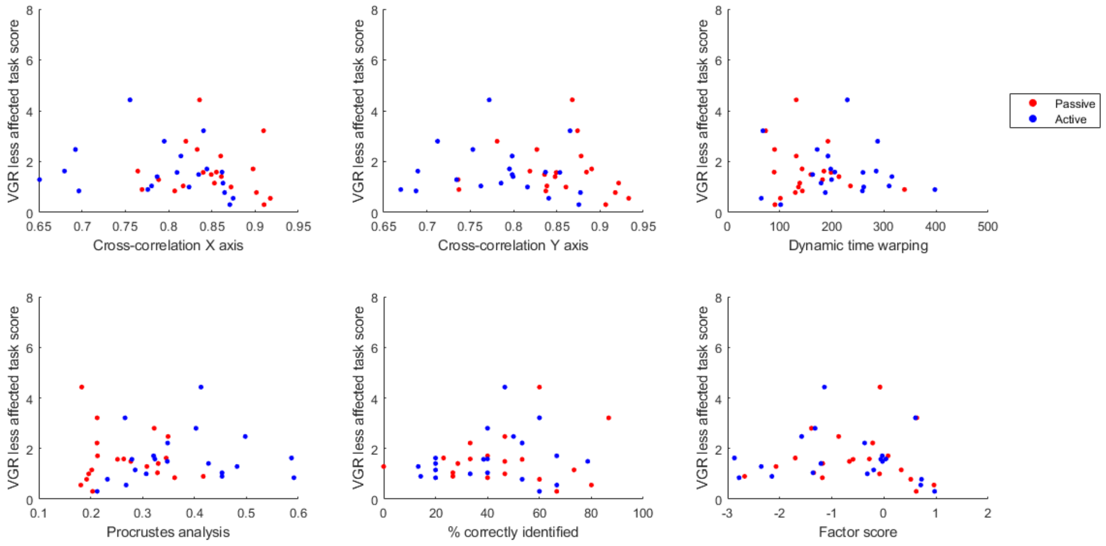

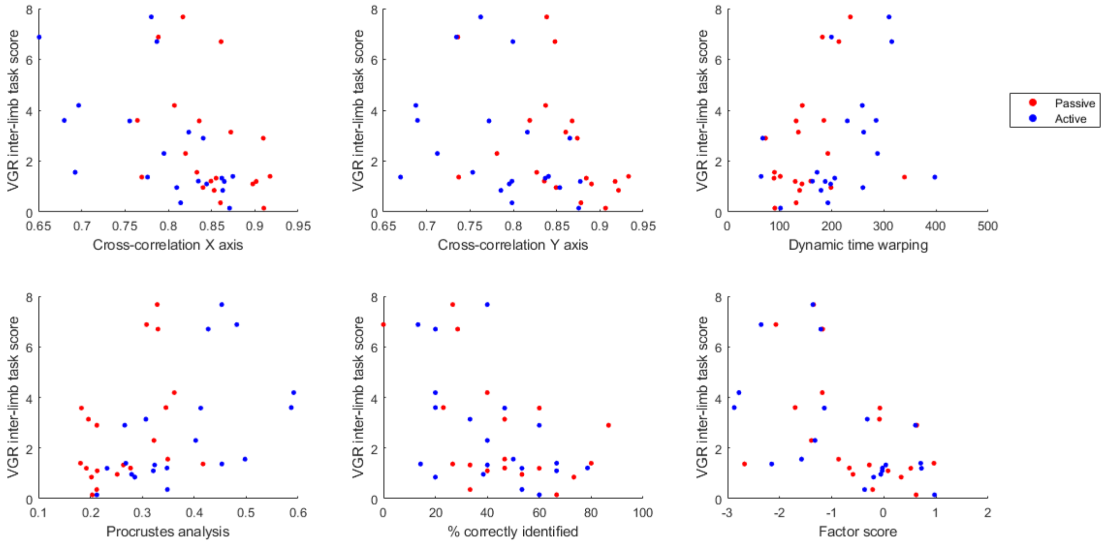

Montreal cognitive assessment

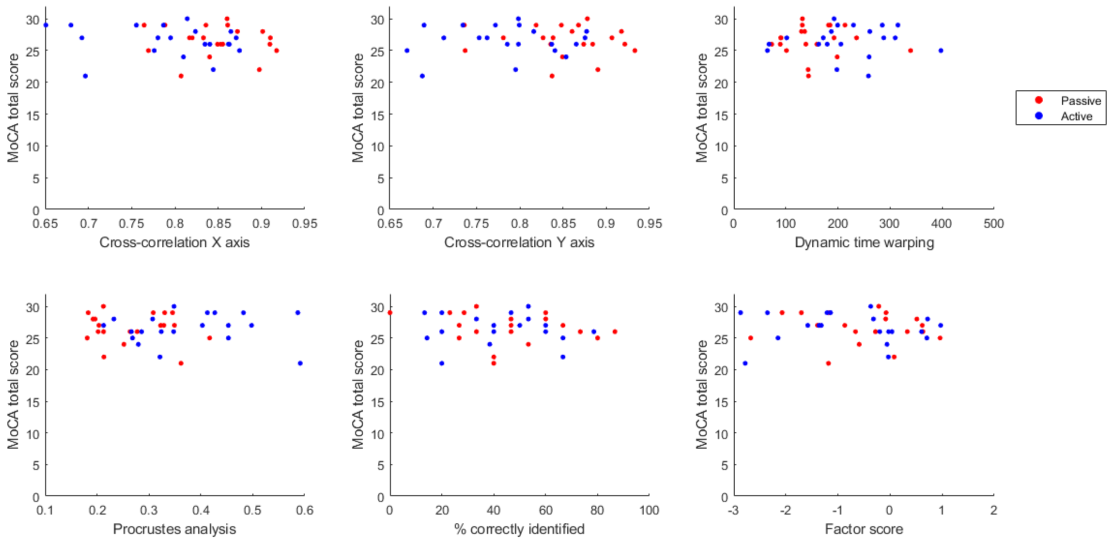

Action research arm test

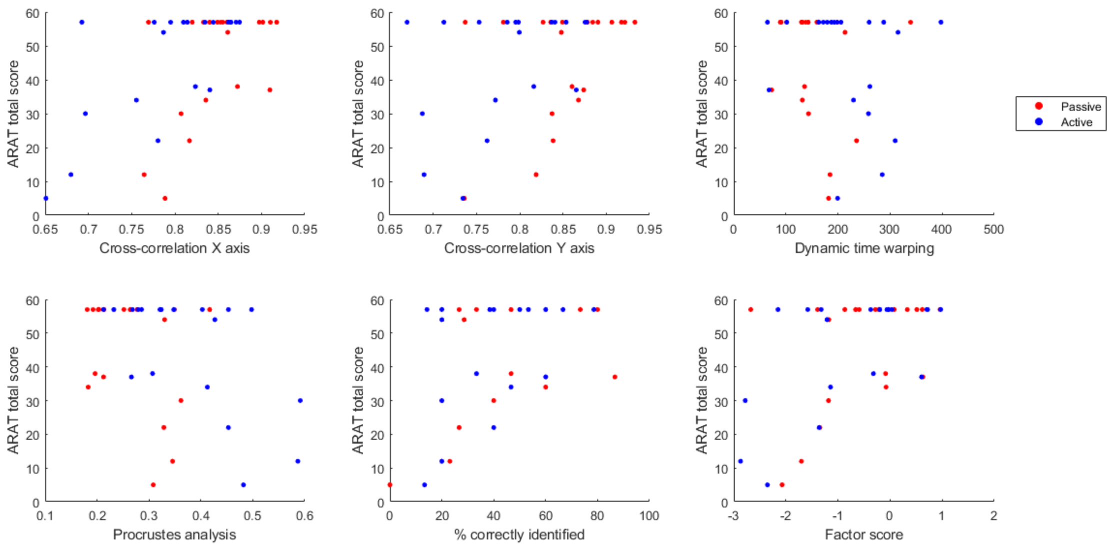

Barthel index

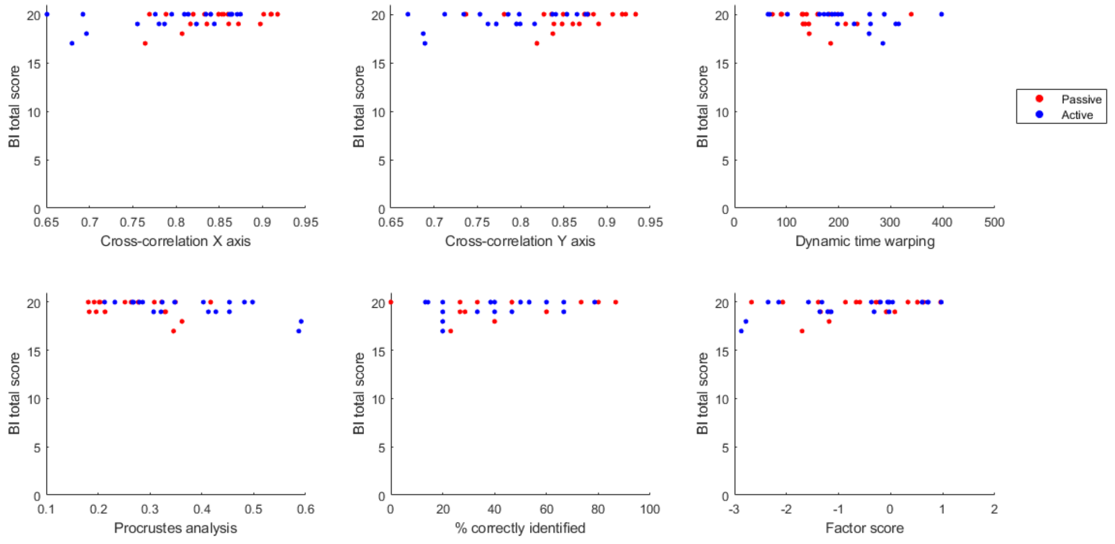

S1: Learning effects for the robot-based passive and active sensory processing tasks.

The presence of a learning effect was evaluated for all reproduction and identification parameters and for the factor score through all 30 trials by performing a robust regression based on a modified bootstrap method of ordinary least squares regression (using the 'lsfitci' function from Wilcox 2017 [44]). Standardized regression coefficients were calculated, and a learning effect was not considered present when the 95% confidence interval of the standardized regression coefficients contained zero.

Cross-correlation on the X axis, Procrustes analysis and factor scores did not show learning effects as shown with a 95% confidence interval of −0.022 to 0.059, 0.058 to 0.026 and −0.022 to 0.064 for the standardized regression coefficients, respectively. Cross-correlation on the Y axis, dynamic time warping and percentage of identified shapes showed only negligible learning effects as shown with a 95% confidence interval of 0.000 to 0.079, −0.095 to −0.012 and 0.013 to 0.095 for the standardized regression coefficients, respectively.

S2: Subgroup analysis to compare performance on the robot-based passive and active sensory processing tasks between participants with stroke with and without clinical sensory processing deficits.

Participants with stroke were classified as having clinical sensory processing deficits if they presented with a score of 7 or lower out of 8 on the sharp-blunt discrimination subscale of the Erasmus modified Nottingham sensory assessment [1].

Cross-correlation values were compared using a robust three-way ANOVA based on 20% trimmed means (‘bwwtrim’ from Wilcox 2017 [44]) using participant subgroup (participants with stroke with vs. without clinical sensory processing deficits) as a between-subject factor, and task condition (active vs. passive) and axis direction (X vs. Y) as the within-subject factors. Dynamic time warping, Procrustes analyses, the percentage of identified shapes and factor scores were compared using a robust two-way ANOVA based on 20% trimmed means (‘bwtrim’ from Wilcox 2017 [44]), with the participant subgroup as the between-subject factor and task condition as the within-subject factor. When no interaction effect was present, we reported the main effects. When an interaction effect was significant, the simple main effects were evaluated. We corrected for multiple comparisons using the Holm–Bonferroni method whenever simple main effects were calculated (‘p.adjust’ from the stats package [41]) [45]. Effect sizes were reported as generalized eta squared [46,47] (‘anova_summary’ from the rstatix package [48]).

Participants with stroke who presented with clinical sensory processing deficits showed worse but not significantly different shape reproduction than participants with stroke who did not present with clinical sensory processing deficits.

For cross-correlation, the subgroup difference was significantly influenced by task condition (Fig. 1A; subgroup x condition: $F(1,12) = 6.20$, $p = 0.013$, $\eta^2_G = 0.02$). For the passive task,

cross-correlation values were worse but not significantly different for participants with stroke who showed clinical sensory processing deficits in comparison to participants without clinical sensory processing deficits (Fig. 1A; $F(1,12) = 1.42$, $p = 0.170$). Participants with stroke who showed clinical sensory processing deficits showed a mean cross-correlation value of 0.84 (SD 0.05) on the passive task, while participants with stroke who did not show clinical sensory processing deficits showed a mean of 0.86 (SD 0.05). For the active task, we found a significant difference between both subgroups (Fig 1A; $F(1,12) = 2.63$, $p = 0.033$). Participants with stroke who showed clinical sensory processing deficits showed a mean cross-correlation value of 0.76 (SD 0.07) on the active task, while participants with stroke who did not show clinical sensory processing showed a mean of 0.81 (SD 0.05).

For dynamic time warping, we found no significant difference between both participant subgroups (Fig. 1B; main effect of subgroup: $F(1,16) = 1.72$, $p = 0.217$, $\eta^2_G = 0.04$). Participants with stroke who showed clinical sensory processing showed a mean score of 202.43 (SD 64.23), while participants with stroke who did not show clinical sensory processing showed a mean score of 172.49 (SD 88.99). There was no evidence that the subgroup difference was influenced by task condition (Fig. 1B; subgroup x condition: $F(1,16) = 1.12$, $p = 0.312$, $\eta^2_G = 0.02$). For the Procrustes analysis, participants with stroke who showed clinical sensory processing deficits showed worse values than participants with stroke who did not show clinical sensory processing deficits, but the difference was not significant (Fig. 1C; main effect of subgroup: $F(1,16) = 4.45$, $p = 0.064$, $\eta^2_G = 0.17$). Participants with stroke who presented with clinical sensory processing deficits showed a mean score of 0.36 (SD 0.12), while participants with stroke who did not show clinical sensory processing showed a mean score of 0.29 (SD 0.08). No evidence was found that the subgroup difference was influenced by task condition (Fig. 1C; subgroup x condition: $F(1,16) = 2.39$, $p = 0.148$, $\eta^2_G = 0.03$).

Participants with stroke who showed clinical sensory processing deficits identified less shapes than participants with stroke who did not show clinical sensory processing deficits.
During the identification phase, participants with stroke who showed clinical sensory processing deficits identified less shapes than participants with stroke who did not show clinical sensory processing deficits (Fig. 1D; main effect of subgroup: $F(1,16) = 4.49$, $p = 0.056$, $\eta^2_G = 0.16$). Participants with stroke who showed clinical sensory processing deficits identified on average 34.91% (SD 17.42), while participants with stroke who did not show clinical sensory processing deficits identified on average 50.82% (SD 19.76). There was no evidence that the

subgroup difference was influenced by task condition (Fig. 1D; subgroup x condition: $F(1,16) = 0.10$, $p = 0.761$, $\eta^2_G = 0.01$).

Stroke patients with clinical sensory processing deficits showed worse sensory processing ability than patients without clinical sensory processing deficits, with a medium effect size shown.

Participants with stroke who did not show clinical sensory processing deficits showed a mean factor score of −0.29 (SD 0.97), while participants with stroke who did show clinical sensory processing deficits showed a worse mean factor score of −1.09 (SD 1.05), meaning that participants with stroke who did show clinical sensory processing showed a worse sensory processing ability (Fig. 1E; main effect of subgroup: $F(1,16) = 4.33$, $p = 0.062$, $\eta^2_G = 0.14$). There was no evidence that the group difference was influenced by task version (Fig. 1E; interaction effect between subject group and task version: $F(1,16) = 1.99$, $p = 0.184$, $\eta^2_G = 0.02$).

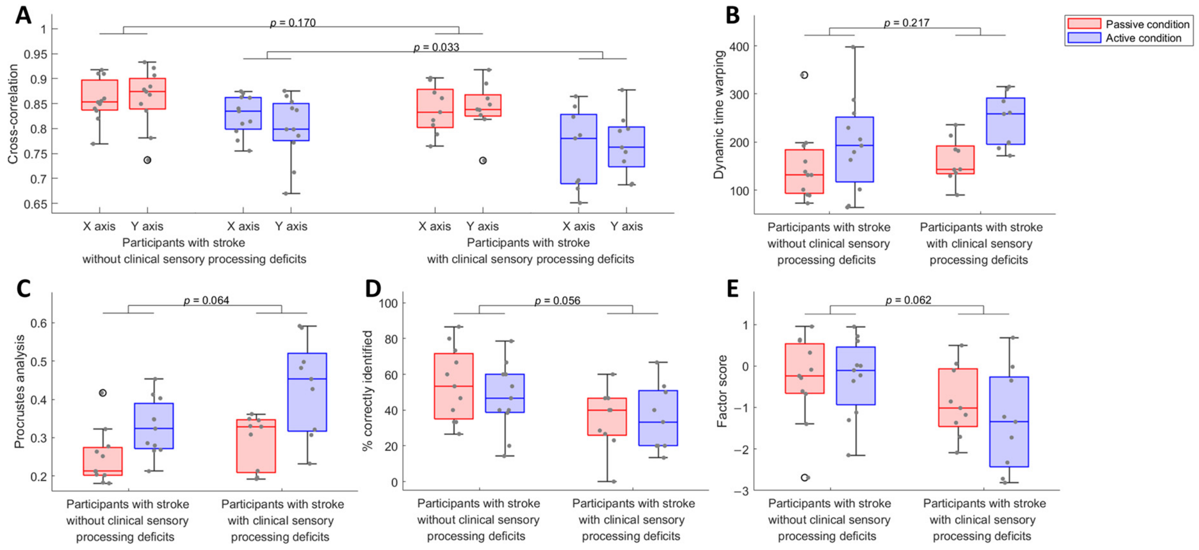

Figure S3. Results of the passive (in red) and active (in blue) sensory processing assessments. **A.** Two-way interaction effects for between-subgroup analysis of three-way ANOVA for cross-correlation on X and Y axes. **B.** Main effect for between-subgroup analysis of two-way ANOVA for dynamic time warping. **C.** Main effect for between-subgroup analysis of two-way ANOVA for Procrustes analysis. **D.** Main effect for between-subgroup analysis of two-way ANOVA for the percentage of correctly identified shapes. **E.** Main effect for between-subgroup analysis of two-way ANOVA for the factor score.

References

1. Recovery after Stroke: Long Term Outcome and Upper Limb Somatosensory Function. (Doctoral thesis, KU Leuven, Leuven, Belgium). Retrieved from https://limo.libis.be/permalink/f/t5q1v2/LIRIAS1687925

41. R Core Team R: A Language and Environment for Statistical Computing; R Foundation for Statistical Computing: Vienna, Austria, 2020.

44. Wilcox, R.R. Introduction to Robust Estimation and Hypothesis Testing, 4th ed.; Elsevier: Amsterdam, The Netherlands, 2017; ISBN 9780128047330.

45. Holm, S. A Simple Sequentially Rejective Multiple Test Procedure. Scand. J. Stat. 1979, 6, 65–70.

46. Bakeman, R. Recommended Effect Size Statistics for Repeated Measures Designs. Behav. Res. Methods 2005, 37, 379–384. https://doi.org/10.3758/BF03192707.

47. Lakens, D. Calculating and Reporting Effect Sizes to Facilitate Cumulative Science: A Practical Primer for t-Tests and ANOVAs. Front. Psychol. 2013, 4, 863. https://doi.org/10.3389/fpsyg.2013.00863.

48. Kassambara, A. Rstatix: Pipe-Friendly Framework for Basic Statistical Tests. Available online: https://rpkgs.datanovia.com/rstatix/ (accessed on 13 February 2021).
